# Molecular Crosstalk Between Circadian Rhythmicity and the Development of Neurodegenerative Disorders

**DOI:** 10.3389/fnins.2020.00844

**Published:** 2020-08-06

**Authors:** Arastu Sharma, Sehyun Lee, Hoonseo Kim, Hargsoon Yoon, Shinwon Ha, Sung Ung Kang

**Affiliations:** ^1^Neuroregeneration and Stem Cell Programs, Institute for Cell Engineering, Johns Hopkins University School of Medicine, Baltimore, MD, United States; ^2^Neural Engineering and Nano Electronics Laboratory, Department of Engineering, Norfolk State University, Norfolk, VA, United States

**Keywords:** circadian rhythm, DNA damage, PARP1, PAR-dependent E3 ligase, α-synuclein pre-formed fibrils, β-amyloid peptides, Parkinson’s disease, Alzheimer’s disease

## Abstract

Neurodegenerative disorders have been shown to exhibit substantial interconnectedness with circadian rhythmicity. Alzheimer’s patients exhibit high degradation of the suprachiasmatic nucleus (SCN), the central endogenous circadian timekeeper, and Parkinson’s patients have highly disrupted peripheral clock gene expression. Disrupted sleep patterns are highly evident in patients with neurodegenerative diseases; fragmented sleep has been shown to affect tau-protein accumulation in Alzheimer’s patients, and rapid eye movement (REM) behavioral disorder is observed in a significant amount of Parkinson’s patients. Although numerous studies exist analyzing the mechanisms of neurodegeneration and circadian rhythm function independently, molecular mechanisms establishing specific links between the two must be explored further. Thus, in this review, we explore the possible intersecting molecular mechanisms between circadian rhythm and neurodegeneration, with a particular focus on Parkinson’s disease. We provide evidence for potential influences of E3 ligase and poly adenosine diphosphate (ADP-ribose) polymerase 1 (PARP1) activity on neurodegenerative pathology. The cellular stress and subsequent DNA damage signaling imposed by hyperactivity of these multiple molecular systems in addition to aberrant circadian rhythmicity lead to extensive protein aggregation such as α-synuclein pre-formed fibrils (α-Syn PFFs), suggesting a specific molecular pathway linking circadian rhythmicity, PARP1/E3 ligase activity, and Parkinson’s disease.

## Molecular Machinery of Circadian Rhythmicity

Living systems on earth are governed by many natural laws, but circadian rhythms play one of the most important roles in sustaining organisms, acting as the biological timekeepers that perpetuate life from mere seconds to the full Gregorian year. Mammalian circadian rhythms can be observed from the genetic level to the tissue level, and even to the macroscopic level, affecting behavior, biochemical and physiological processes. Genetic and cellular clocks dictate *tau* (free running period of the organism ∼24 h) and are often entrained by photo-optic cues (Dunlap and Loros, 2017). Light, as the external stimulus, activates intrinsically photosensitive retinal ganglion cells (ipRGCs), which innervate the suprachiasmatic nucleus (SCN) in mammals, entraining the mammal to the 24-h day (Do and Yau, 2010). The SCN assumes the role as the central pacemaker, and through a series of genetic feedback loops and highly coordinated neuronal innervation, endogenous timekeeping activity arises, giving way to the production of circadian rhythm. To garner a more comprehensive understanding of the effects of circadian rhythms on health and neurodegeneration, the underlying fundamental molecular mechanisms and interrelated processes must be explored (Cox and Takahashi, 2019).

**FIGURE 1:**
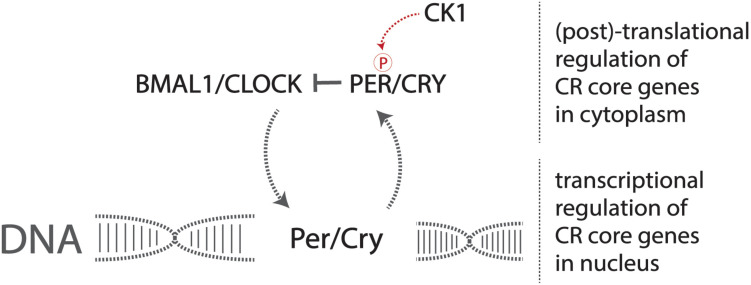
The core circadian molecular machinery gives rise to endogenous timekeeping activity. The CLOCK/BMAL1 heterodimer activates the transcription of *Per/Cry* genes, and the production and resulting phosphorylation of PER/CRY inhibit the CLOCK/BMAL1 heterodimer, reducing the transcription of *Per/Cry* forming the negative feedback loop required to maintain circadian rhythmicity at a basic molecular level.

A set of core genes constitutes this transcriptional pathway that forms the identity of the endogenous circadian pacemaker. Two heterodimerization transcription factors, CLOCK and BMAL1, activate the production of the period (*Per*) and cryptochrome (*Cry*) genes (Yoo et al., 2005; Figure 1). The resulting PER and CRY proteins heterodimerize and are phosphorylated by casein kinase 1 (CK1), allowing for translocation into the nucleus, consequently inhibiting CLOCK and BMAL1 transcriptional activity, preventing the production of PER and CRY, forming the central negative feedback loop that gives rise to endogenous circadian rhythmicity at the most basic cellular level in mammals (Philpott et al., 2020). Mutations in these genes result in altered displays of circadian behavior in affected model organisms (Patke et al., 2020).

Irregularities in circadian rhythms have been shown to alter sleep–wake cycles, as well as alter metabolic processes that may result in the development of certain diseases or other health-related complications like diabetes, cardiovascular disease, cancer, and neurodegenerative disorders (Wilking et al., 2013; Qian and Scheer, 2016).

## Circadian Rhythmicity in the Context of Neurodegenerative Pathways

**FIGURE 2:**
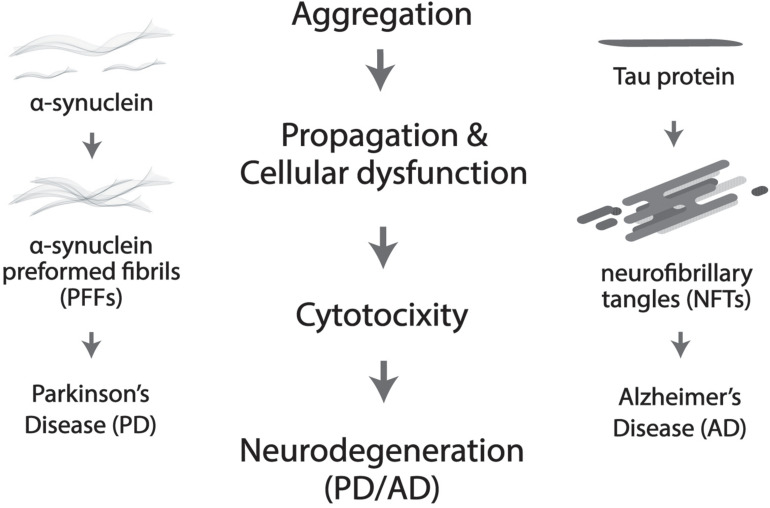
Protein aggregation results in cellular dysfunction and cytotoxicity, which further leads to neurodegeneration. More specifically, neurofibrillary tangles (NTFs) caused by Tau aggregation is the culprit in Alzheimer’s disease (AD) and α-synuclein pre-formed fibrils (α-Syn PFFs) by α-synuclein accumulation in Parkinson’s disease (PD).

More prevalent in old age, neurodegenerative disorders such as Alzheimer’s disease (AD) and Parkinson’s disease (PD) are widely believed to be driven by protein deposition in the form of insoluble aggregates, resulting in loss of physiological functions and pathological dysregulation of neurons (Delenclos et al., 2019; Huseby et al., 2019; Figure 2). Severe degradation of the SCN, as well as negatively altered patterns of circadian expression are prevalent in AD patients, but in PD much more targeted degradation of dopaminergic (DA) striatal neurons occurs (Dauer and Przedborski, 2003). Lower levels of BMAL1 are directly proportional to the gravity of an individual’s development of PD (Cai et al., 2010; Ding et al., 2011). Melatonin treatment was shown to prevent DA neuronal cell degradation, but the mechanism through which the protection of DA neurons occurs is unclear, whether it be oxidative or circadian (Radogna et al., 2010; Wilking et al., 2013).

Circadian irregularities can also be symptomatic and result in comorbidities, such as cases of individuals with rapid eye movement (REM) behavioral disorder that will ultimately develop PD (Breen et al., 2014). Sleep serves a critical role in the maintenance of circadian rhythms and can strongly affect the buildup of neuronal stress (Breen et al., 2014). In the case of AD, β-amyloid peptide (Aβ) plaques build up in neurons, causing severe systemic stress and eventually leading to the destruction of the cell (Kocahan and Dogan, 2017). Aβ accumulation occurs from natural activity of the cell, and fluctuates with circadian rhythmicity, with higher levels apparent during waking hours and drop-offs during periods of inactivity (Kang et al., 2009).

Alzheimer’s disease models have exhibited disrupted patterns of glymphatic flow (GF) in rodents, and GF has also been shown to be regulated by sleep activity, where slow wave sleep in mice resulted in a 60% increase in interstitial brain fluid and increased GF. This phenomenon is crucial for the removal of excess molecular buildup, particularly Aβ, in the extracellular space of areas with high neuronal activity in the central nervous system (Rasmussen et al., 2018). A vital protein associated with Aβ plaque aggregation and tau protein pathology is Orexin, a compound involved in the sleep–wake pathway that promotes wakefulness. Through analyzing its activity, researchers found that cyclic levels of orexin during normal rhythmic activity were inversely proportional to tau protein aggregation; high Aβ plaque aggregation and tau protein pathology also result in disturbances in non-REM sleep activity, ascertaining the bidirectional influence of circadian rhythmicity and AD pathology (Kilduff et al., 2008; Kang et al., 2009; Liu et al., 2019). Multiple studies found that reduced wakefulness as a result of orexin inhibition reduced levels of Aβ buildup in cells (Davies et al., 2015; Liu et al., 2019). The SCN of affected AD patients was also found to have experienced severe degeneration, and clock gene expression in various regions of the brain was highly asynchronous.

Parkinson’s disease patients exhibit asynchronous peripheral clock gene expression, therefore single nucleotide polymorphisms in various clock genes have been proposed to indicate the risk of PD development in affected individuals (Gu et al., 2015). Patients affected with PD exhibit much more flattened circadian diurnal curves as a result of increased activity during inactive hours and decreased activity during waking hours (Porter et al., 2008; Verbaan et al., 2008). The majority of PD patients suffer from sleep disturbance as one of the primary symptoms, most likely a result of severe degeneration of the raphe nucleus and locus coeruleus (Braak and Del Tredici, 2008). Circadian melatonin rhythmicity is also altered in PD patients, with the curve being flattened over the course of 24 h (Videnovic and Golombek, 2017).

**FIGURE 3:**
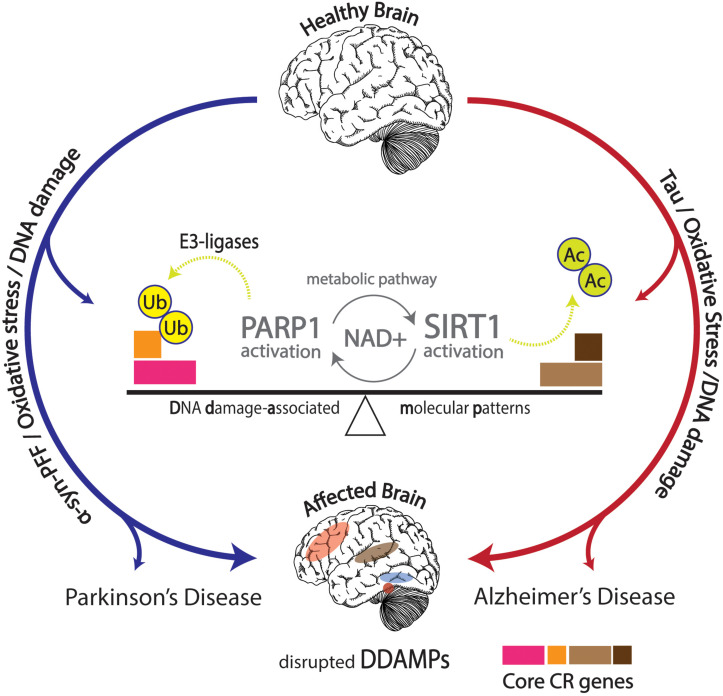
Effects of DNA damage in conjunction with α-synuclein pre-formed fibrils (α-Syn PFFs) and tau pathology shown in the affected brain with disrupted DNA damage-associated molecular patterns (DDAMPs) in Parkinson’s disease (PD) and Alzheimer’s disease (AD), respectively. Disruptions in metabolic pathways lead to altered DNA repair mechanisms, and subsequent poly (ADP-ribose) polymerase 1 (PARP1) and E3 ligase activity, leading to the ubiquitination of core circadian genes in particular, further influencing the course of PD pathology in terms of α-Syn PFFs formation and propagation.

Numerous articles of evidence discovered strongly accentuate the interconnected activity between circadian rhythms, metabolic and molecular pathways, aging, diseases, as well as neurodegenerative disorders. Aberrant normal pathological function in the form of neurodegenerative effects has been discussed, but proper ubiquitination of proteins offers stark neuroprotection against development of neurodegenerative disorders characteristic of protein aggregation. Proteasomal activity has been shown to follow circadian rhythmicity, and distortions in this process may result in increased risks of developing neurodegenerative diseases (Musiek and Holtzman, 2016; Figure 3).

## The Effect of Antagonistic E3 Ligases on Circadian Period Length

Functional protein families such as kinases, E3 ligases, (de)acetylases and poly adenosine diphosphate (ADP-ribose) polymerases can directly or indirectly affect patterns of circadian behaviors through post-translational modifications of core proteins. Ubiquitination of PER and CRY proteins is a vital step in maintaining the integrity of the cellular circadian clock. The 26S proteasome uses a series of enzymes such as E3 ligases like FBXL3 and FBXL21 to regulate the degradation of PER, CRY, and other proteins, resulting in disinhibition of CLOCK and BMAL1 transcriptional factors, allowing for the molecular circadian clock to reset and begin the negative feedback loop once again (Nandi et al., 2006). Moreover, there are numerous studies that highlight the relationship between other circadian rhythm proteins and E3 ligases (Table 1).

**TABLE 1:** Circadian rhythm proteins shown to be associated or dependent on E3 ligase activity.

Circadian rhythm Gene	E3 Ligase	Source
CRY1/2	FBXL3	Busino et al. (2007); Godinho et al. (2007), Siepka et al. (2007); Yoo et al. (2013)
CRY1/2	FBXL21	Dardente et al. (2008); Hirano et al. (2013), Yoo et al. (2013)
PER1	β-TRCP1 (FBW1A), β-TRCP2 (FBW1B)	Shirogane et al. (2005)
PER2	β-TRCP1 (FBW1A), β-TRCP2 (FBW1B)	Eide et al. (2005); Reischl et al. (2007), Ohsaki et al. (2008)
REV-ERBα	HUWE1 (ARF-BP1), PAM (MYCBP2)	Yin et al. (2010)
BMAL1	UBE3A	Gossan et al. (2014)
TIM (*Drosophila*)	JETLAG	Naidoo et al. (1999); Koh et al. (2006)
CRY	CG17735, CG11321, and CG5604 (HECT or RING domain-containing E3 ligases), Bruce (E2–E3 ligase)	Sathyanarayanan et al. (2008)
CLOCK/PERIOD	CTRIP	Lamaze et al. (2011)
PER	SUPERNUMERARY LIMBS	Chiu et al. (2008)
PER2	MDM2	Liu et al. (2018)
CRY1	CUL4-DDB1-CDT2	Tong et al. (2015)
Unknown	UBR4 (ubiquitin protein ligase E3 component N-recognin 4) found as a time-of-day-dependent and light-inducible protein	Ling et al. (2014)

Furthermore, recent studies have provided strong evidence confirming the link between metabolic processes, neurodegenerative disorders, and circadian dysfunction, through a focus on the E3 ligase pathways, namely mitochondrial ubiquitin ligase 1 pathway (*Mul1*) and *Parkin* pathways. *Mul1* mutations have been shown to lead to PD symptoms in a similar fashion as *Parkin*/*Pink1* mutations, the usual culprits of the neurodegenerative disorder (Yun et al., 2014). *Mul1* and *Park1* mutations resulted in shorter lifespans and longer amounts of physical activity during a given 24-h period (Doktor et al., 2019). *Mul1* mutants showed a disrupted expression pattern of *Per, Tim*, and *Clock* mRNA compared to wild-type flies, and *Mul1* mutants exhibited a severe drop-off in ATG5 compared to *Park1* mutants and controls (Doktor et al., 2019). ATG5 and other related proteins are critical in autophagy; these proteins form complexes and drive clearance of protein aggregation universally characteristic of neurodegenerative disorders (Doktor et al., 2019).

**FIGURE 4:**
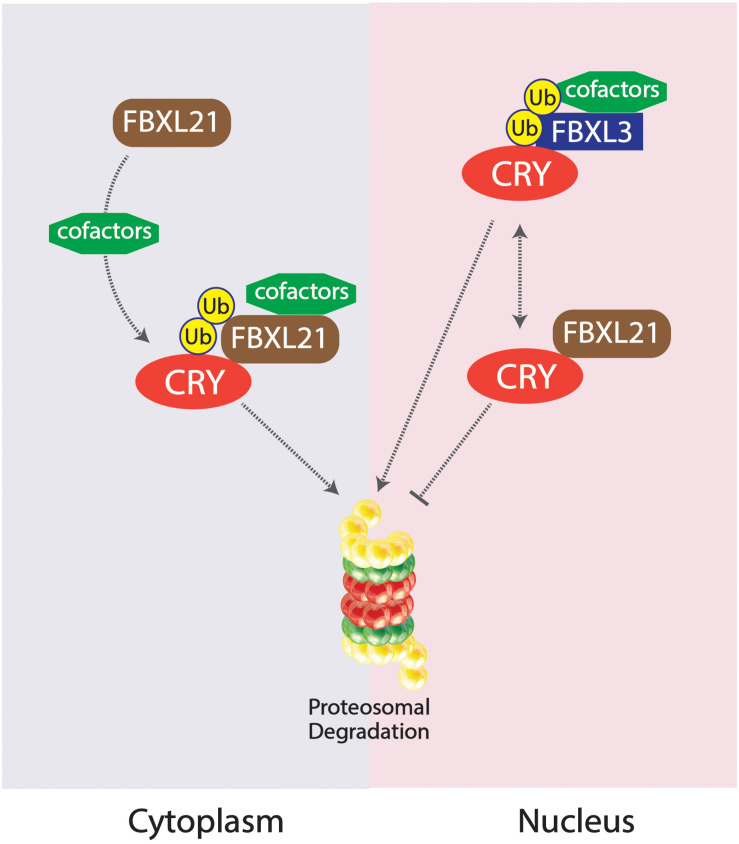
Competing SKP1-Cul1-F-box protein (SCF) E3 ligase complexes SCFFBXL21 and SCFFBXL3 and their effects on CRY degradation in the cytoplasm vs. nucleus. SCFFBXL21 degrades CRY in the cytoplasm but exhibits protective abilities in the nucleus, contrary to the degradative properties of SCFFBXL3 on the CRY protein within the nucleus.

Competing E3 ligase mechanisms involved with CRY ubiquitination have been shown to affect tau in mice. Missense mutations in the *Fbxl*21 gene, the gene expressing a protein which targets CRY for ubiquitination, results in shortened circadian periods due to an accelerated degradation of the CRY protein in the nucleus and the cytoplasm, whereas alterations in the competing ligase FBXL3 results in a longer circadian period (Busino et al., 2007; Godinho et al., 2007; Siepka et al., 2007; Yoo et al., 2013). The duality of these two E3 ligases works to preserve the balance of CRY degradation in the cytoplasm and the nucleus (Yoo et al., 2013). An SKP1-Cul1-F-box protein (SCF) E3 ligase complex is formed constituting FBXL3, targeting CRY proteins for proteasomal degradation; FBXL21 has been inferred to exist as a clock-controlled E3 ligase concerning ovine CRY1 degradation (Siepka et al., 2007; Dardente et al., 2008). The existing mutation Overtime (*Ovtm*) in the FBXL3 protein exists as the functionally antagonistic counterpart to the *Psttm* mutation in FBXL21 by lengthening the period rather than shortening (Siepka et al., 2007; Yoo et al., 2013). Due to the diminished presence of CRY as a result of the *Psttm* mutation in the *Fbxl*21 gene, inhibition of the CLOCK:BMAL1 transcriptionally enhancing complex is further diminished, leading to an increase of RNA expression of target genes. The accelerated degradation of CRY1 is a result of the reduced levels of FBXL21 protein levels. CRY1 was shown to be polyubiquitinated in the presence of the SCFBXL3 complex, but the presence of SCFFBXL21 and SCFPSTTM significantly reduced the ubiquitination of CRY1 by SCFFBXL3, enhancing the antagonistic interactions between FBXL3 and FBXL21. FBXL21 and *Psttm* have been shown to prevent CRY1 degradation due to FBXL3 as well, further exemplifying the competing characteristics between the two E3 ligases. FBXL21’s specificity for CRY1 degradation may hint to the reduced rate at which it ubiquitinates the CRY1 protein, contrasting from the rather liberal utilization of lysine residues by FBXL3 for CRY1 degradation. The fundamental importance of the family of CRY proteins in regulating circadian period length has been fully emphasized through the analysis of competing E3 ligases FBXL21 and FBXL3 mutations. The “paralogous” nature of FBXL21 and FBXL3 interestingly produced antagonizing behavior in the two E3 ligases, competing for the regulation of CRY1 degradation. The preferential interactions between FBXL21 and CRY may support the finding that FBXL21 is able to protect CRY degradation from SCFFBXL3 activity within the nucleus, accentuating the role of E3 ligases in regulating and dictating circadian period through complex molecular interactions within the nucleus (Yoo et al., 2013; Figure 4).

## Poly [ADP-Ribose] Polymerase 1 Activity and Its Influence on Circadian Rhythmicity

The cyclic influence of core circadian proteins on nicotinamide phosphoribosyltransferase/nicotinamide adenine dinucleotide (NAMPT/NAD) + activity ultimately relates to a vital protein: Poly (ADP-ribose) polymerase 1 (PARP1), a nuclear protein responsible for facilitation of DNA strand repair. It is activated in response to single-strand breaks within DNA, attaching a poly ADP-ribose (PAR) chain for subsequent repair by DNA ligases. After the process nears completion, poly ADP-ribose glycohydrolase (PARGs) degrade the remaining attached PAR chain (Herceg and Wang, 2001). The interwoven nature of the mechanisms between PARP1 and NAD + activity possibly hint to the involvement in the development and course of neurodegenerative disorders (Martire et al., 2015). In addition to PARP1 being a critical component of the DNA repair mechanism, it is also a critical component of parthanatos, the cell death pathway involved in cellularly destructive diseases such as stroke and PD (David et al., 2009). The accumulation of PAR within the cell due to hyperactivity of PARP1 in response to DNA damage in conjunction with the translocation of mitochondrial-associated apoptosis-inducing factor (AIF) into the nucleus results in activation of the parthanatos pathway, leading to extensive DNA fragmentation and cell death (Wang et al., 2011). PARP1 activation occurs following any magnitude of DNA damage, with the amount of activation proportional to the severity of genetic stress. The activity of PARP1 results in the formation of PAR polymer chains which ultimately becomes highly toxic to the cell (Andrabi et al., 2006).

The high dependency of PARP1 on NAD + availability leads to the depletion of the NAD + surplus, leading to subsequent cellular stress. Administration of NAD + to energetically starved cells, or the reinstatement of glycolytic factors within the glycolysis cycle, have been shown to reverse the effects of NAD + depletion, offering neuroprotection and preventing the activation of the parthanatos pathway (Fatokun et al., 2014). Circadian expression patterns of NAMPT, the rate limiting enzyme of the NAD + salvage pathway, results in a circadian rhythm of NAD + abundance within the cell, giving rise to circadian activity of PARP1 (Nakahata et al., 2009; Ramsey et al., 2009). Opposing duality of PARG and PARP1 processes may contribute to rhythmicity, but studies have shown that rhythmicity of PARP1 auto-ADP-ribosylation is maintained in spite of manipulation of PARG activity (Davidovic et al., 2001). Studies have shown that circadian PARP1 activity is independent of central pacemakers and heavily regulated by feeding (Damiola et al., 2000; Stokkan et al., 2001). Moreover, abundance of NAD + has been shown to not sufficiently account for the circadian activity of auto-ADP-ribosylation of PARP1, at least *in vitro* (Asher et al., 2010). However, unlike PARP1’s endogenous activity, NAD + activity has been shown to be under the influence of the central clock (Ramsey et al., 2009).

Poly (ADP-ribose) polymerase 1 has also been shown to co-immunoprecipitate with CLOCK and BMAL1. PARP1 has the ability to rhythmically affect CLOCK activity, and highly disrupted circadian and rhythmic activity of the CLOCK-BMAL1 dimer is attributed to inhibition of PARP1 activity (Asher et al., 2010). Wild type CLOCK and BMAL1 forms have exhibited circadian rhythmicity when binding to *Per2* promoters, whereas PARP1 knockout mice exhibited much higher levels of CLOCK and BMAL1 activity. PARP1 knockout and wild type animals exposed to arrhythmic feeding patterns did not exhibit altered differences in genetic expression, but PARP1 knockout mice exhibited a lower level of mRNA of target genes of CLOCK and BMAL1. Additionally, PARP1 knockout mice exhibited a lengthened Tau compared to wild-type mice, suggesting that direct consequences of PARP1 activity could possibly manifest in the SCN (Asher et al., 2010).

## Alteration of PAR-Dependent E3 Ligase Activity on Parkinson’s Disease in Response to DNA Damage

Another highly apparent interaction between E3 ligases, DNA repair and PARP1 activity has been portrayed through the E3 ligase, Iduna. Iduna’s really interesting new gene (RING) finger domain’s zinc binding activity is responsible for its E3 ligase activity, and mutations in the H54A and C60A domains result in absence of ubiquitination activity. The Iduna YRAA mutant disrupts the PAR-binding abilities, and the autoubiquitination abilities of Iduna’s E3 ligase activity have been shown to be directly dependent on PAR concentrations. Two of the primary targets of Iduna have been shown to be PARP1 and PARsylated PARP1. The presence of proteasomal inhibitors, such as MG132, has been shown to inhibit Iduna’s ability to degrade PARP1 and PARsylated PAR, affirming the ubiquitinated PAR-dependent degradation of PARP1 by Iduna. Microirradiation induced DNA damage resulted in an eightfold increase in the amount of apurinic/apyrimidinic sites. The prevention of this increase is possible through overexpression of Iduna. However, Iduna YRAA mutants were unable to suppress the increase of apurinic/apyrimidinic sites due to the lack of PAR binding ability, showing that PAR-binding is crucial for suppression of genetic lesions. Iduna C60A mutants also failed to reduce the increase in DNA lesions, emphasizing that the E3 ligase activity of Iduna is also crucial for DNA maintenance and repair. These complex interactions between DNA damage, PAR-related activity, and E3 ligase activity begin to paint a picture of highly interrelated intricacy, affirming that Iduna acts as a PAR-dependent ubiquitin E3 ligase. Iduna has also been shown to protect the brain from stroke by disruption of the parthanatos mechanism (Kang et al., 2011).

The earlier explored interactions allow for the analysis of the effects of PARP1 activity and subsequent PAR accumulation on the development and course of PD. Accumulation of α-Syn PFFs has been shown to activate PARP1, leading to the generation of PAR aggregates, compounding into large combined aggregates that become toxic for given cells, eventually resulting in cell death via parthanatos (Kam et al., 2018). After administration of α-Syn PFFs in mice, higher PARP1 activity was observed for a period of 2 weeks, whereas administration of PAR inhibitors prevented α-syn PFFs induced cell death. Deletion of PARP1 through CRISPR similarly resulted in prevention of α-syn PFFs induced PARP1 activity, cell death, α-Syn aggregation, as well as cell-to-cell α-Syn PFFs transmission. Nitric oxide (NO) synthesis was also increased after α-syn PFFs administration, whereas NO inhibitors prevented this activation. After administration, increased PAR levels and PARP1 activity were observed, but PAR levels were not increased due to the absence of PARP1 in PARP1 knockout mice, and can be attributed to the fact that PAR aggregation is a result of PARP1 activity. Parthanatos in DA neurons was observed after administration in wild-type mice, but loss of DA neurons was not observed in PARP1 knockout mice and in mice previously injected with ABT-888, a PARP1 inhibitor. PAR has been shown to directly increase the rate of aggregation of α-Syn PFFs in neuronal cells, and 20% of PAR binding to these fibrils was observed in the mouse brain. PAR has also been shown to produce a toxic strain of α-Syn PFFs in the form of more compact and misfolded aggregation. The highly accelerated initial degradation of DA neurons *in vivo* can be attributed to the presence of PAR α-Syn PFFs rather than the generic species of α-Syn PFFs. PD patients have also exhibited higher levels of PAR in their cerebrospinal fluid as well as in the substantia nigra (Kam et al., 2018). The link between PARP1 and E3 ligase activity, the affected pathology in PD, and underlying circadian processes all ultimately point to a highly interrelated mechanism, implying the possibilities of circadian treatment as a preventative and treatable measure for PD.

## Conclusion

The complex web of molecular interactions points to the possibility of much more entangled pathological similarities between PD pathology, DNA damage and repair, and the endogenous regulation of core circadian genetic elements through E3 ligase and PARP1 activity. This specific targeting of PARP1 in respect to PD treatment exhibits mirrored similarity to the mechanism through which SIRT1 is targeted in AD treatment (Wong and Tang, 2016). The interconnectedness between circadian rhythmicity, protein aggregation, and metabolic activity is exemplified by PARP1, suggesting its role as a therapeutic target in treatment for aggressive progression of PD (Figure 3).

Circadian irregularities have been shown to have bi-directional physiological and metabolic implications on general health, as well as influence on and influence from neurodegenerative disorders. E3 ligase and PARP1 activity are essential in regulating both circadian rhythmicity and proper neuronal function. Thus, the dysregulation of these pathways have the capability of causing cellular stress and DNA damage that could further lead to neurodegenerative diseases like AD and PD. DNA damage response (DDR) has been shown to be reliant on the circadian properties of the cell through previously explored mechanisms of PARP1 and E3 ligases. Aberrant circadian qualities may disrupt the DDR or in turn be disrupted by hyperactivity of PARP1 and E3 ligases, ultimately leading to PAR induced accelerated accumulation of neurotoxic strains of α-Syn, further activating PARP1, contributing to the pathogenesis of PD. Regulating proper circadian function may be crucial for preventing the mechanistic onset of molecular complications heralding neurodegenerative distress.

## Author Contributions

AS, SL, and SK designed and planned this review manuscript. AS, SL, HY, and SK wrote the manuscript. AS, SL, HK, SH, and SK contributed to the literature search, collection, and summary. HY provided amendments to draft versions of the manuscript. All authors contributed to the article and approved the submitted version.

## Conflict of Interest

The authors declare that the research was conducted in the absence of any commercial or financial relationships that could be construed as a potential conflict of interest.
